# The Impact of the COVID-19 Pandemic on the Practice of Forensic Medicine: An Overview

**DOI:** 10.3390/healthcare10020319

**Published:** 2022-02-08

**Authors:** Massimiliano Esposito, Monica Salerno, Edmondo Scoto, Nunzio Di Nunno, Francesco Sessa

**Affiliations:** 1 Department of Medical, Surgical and Advanced Technologies “G.F. Ingrassia”, University of Catania, 95121 Catania, Italy; massimiliano.esposito91@gmail.com (M.E.); monica.salerno@unict.it (M.S.); dott.edmondo.scoto@gmail.com (E.S.); 2 Department of History, Society and Studies on Humanity, University of Salento, 73100 Lecce, Italy; nunzio.dinunno@unisalento.it; 3 Department of Clinical and Experimental Medicine, University of Foggia, 71122 Foggia, Italy

**Keywords:** COVID-19, SARS-CoV-2, forensic sciences, pandemic scenario

## Abstract

During the COVID-19 pandemic, forensic sciences, on the one hand, contributed to gaining knowledge about different aspects of the pandemic, while on the other hand, forensic professionals were called on to quickly adapt their activities to respond adequately to the changes imposed by the pandemic. This review aims to clarify the state of the art in forensic medicine at the time of COVID-19, discussing the following: the influence of external factors on forensic activities, the impact of autopsy practice on COVID-19 and vice-versa, the persistence of SARS-CoV-2 RNA in post-mortem samples, forensic personnel activities during the SARS-CoV-2 pandemic, the global vaccination program and forensic sciences, forensic undergraduate education during and after the imposed COVID-19 lockdown, and the medico-legal implications in medical malpractice claims during the COVID-19 pandemic. The COVID-19 pandemic has greatly influenced different aspects of human life, and, accordingly, the practical activities of forensic sciences that are defined as multidisciplinary, involving different expertise. Indeed, the activities are very different, including crime scene investigation (CSI), external examination, autopsy, and genetic and toxicological examinations of tissues and/or biological fluids. At the same time, forensic professionals may have direct contact with subjects in life, such as in the case of abuse victims (in some cases involving children), collecting biological samples from suspects, or visiting subjects in the case of physical examinations. In this scenario, forensic professionals are called on to implement methods to prevent the SARS-CoV-2 infection risk, wearing adequate PPE, and working in environments with a reduced risk of infection. Consequently, in the pandemic era, the costs involved for forensic sciences were substantially increased.

## 1. Introduction

On 9 January 2020, the China CDC (the Centre for Disease Control and Prevention) identified severe acute respiratory syndrome coronavirus 2 (SARS-CoV-2) as the pathogen of coronavirus disease 2019 (COVID-19) [1]. The efforts made by the international scientific community have been remarkable: from the point of view of scientific production, we are witnessing something never seen before, also in view of the fact that on 11 March 2020, the World Health Organization (WHO) declared the COVID-19 outbreak a “pandemic”, assessing the severity levels and global spread of the SARS-CoV-2 infection [2]. To date (3 December 2021), entering the keyword “COVID-19” in the Scopus database shows that 230,109 papers (146,014 original articles) have been published in less than two years; in the same way, entering the keyword “SARS-CoV-2” produces 99,957 papers (63,954 original papers) that match this keyword. Moreover, as analyzed by Haghani and Bliemer [3], the scientometric aspects of the COVID-19 literature involve different research fields (from general medicine to social sciences), demonstrating a vast scientific production worldwide. This unprecedented scientific mobilization has had important results in the battle against COVID-19, such as the biosynthesis of different vaccines.

In this scenario, forensic sciences, on the one hand, contributed to gaining knowledge about different aspects of the COVID-19 pandemic, while on the other hand, forensic professionals were called on to quickly adapt their activities in order to respond adequately to the changes imposed by the pandemic.

This narrative review aims to provide an up-to-date profile on the different aspects that are still ongoing. In particular, this review aims to clarify the state of the art in forensic medicine at the time of COVID-19, discussing the following topics: the influence of external factors on forensic activities, the impact of autopsy practice on COVID-19 and vice-versa, the persistence of SARS-CoV-2 RNA in post-mortem samples, forensic personnel activities during the SARS-CoV-2 pandemic, the global vaccination program and forensic sciences, forensic undergraduate education during and after the imposed COVID-19 lockdown, and the medico-legal implications in medical malpractice claims during COVID-19 pandemic. The methodology approach is reported in the Supplementary Material.

## 2. The sCOVID-19 Pandemic and Its Impact on Forensic Investigations

### 2.1. Influence of External Factors on Forensic Activities

The COVID-19 pandemic radically changed the nature of social interaction and economic activity in all regions of the world [4]. Governments in different countries applied and are still applying a wide range of restrictions, adapting and readjusting their response according to the course of the pandemic [5].

Analyzing national and international data from some countries shows that the unprecedented changes related to the pandemic differ by type of crime, by country or region, and over time. Obviously, if the type of crime committed changes, the countermeasures to prevent crime change accordingly. The polices to contain the spread of infection have altered all habits of life, including criminal activities. In a recent report examining the situation in 27 cities worldwide, the impact of the COVID-19 pandemic on certain types of crime, such as assault, theft, burglary, robbery, vehicle theft and homicide, has been remarkable [6]. The stay-at-home policies have interrupted or reduced the daily movements in time and space of all citizens, generating a reduction in the number of crimes committed. Similar results are seen in the statistical report that analyzes the impact of the COVID-19 pandemic on crime in England and Wales in the period from May 2020 to March 2021 [7]. A reduction of the same type of crimes has been reported in Chicago [8]. These data are confirmed in Argentina, where the quarantine restrictions caused a significant decline in property crime, although no significant changes in numbers of homicides were described [9].

Conversely, there has been an increase in other types of crime, such as domestic violence and cyber fraud that came in the form of ads, emails, fake websites, but also through phone calls and messages [7].

As reported before, in order to slow down the spread of SARS-CoV-2, especially between February–June 2020, many countries adopted the isolation of citizens inside their homes, the so-called lockdown [10]. These measures, on the one hand, controlled the spread of COVID-19, and on the other produced psychological repercussions [11]. During this period, there was an increase in domestic violence (DV). The definition of DV means partner violence [12,13,14,15,16], elder abuse [17], and child abuse [18,19,20] (sexual, physical, psychological violence). In fact, during this period in China DV tripled, in France there was a 30% increase, and in Brazil DV increased by 40/50% [21]. In the UK, femicides doubled between 23 March and 12 April 2020 compared to the previous 10 years [22]. Of course, spending more time in a confined environment such as the home increases the risk of conflict between family members. In addition to this factor, the stress induced by the pandemic also has negative consequences on the economic, social, and psychological aspect of a family. All of this is a major risk factor for DV. Furthermore, confinement induces a fear for victims to report any violence suffered [23]. Several solutions have been proposed to deal with DV during the COVID-19 pandemic, such as increasing the health response, improving police efficiency, and strengthening social safety nets. However, there are some more effective methods such as improving the reporting system of potential DV through TV advertising, and raising awareness among neighbors. The sharing of space negatively affects reports from victims of DV, therefore the possibility of offering these people temporary accommodation could be useful [24]. In Colombia, several measures were proposed to prevent DV. First, health professionals needed to raise awareness of the issue and provide front-line psychological assistance. Secondly, mass awareness was needed through all means of communication (TV, social media, internet). A third measure was to ensure adequate legal action against attackers for cases reported during lockdown [25]. To prevent DV, Campbell formulated the “Opportunity to Abuse Theory”, according to which he proposed to take three measures. The first was to provide reporting cards to all operators who managed to enter the households of others, such as garbage collectors, postmen, and staff who make home deliveries. The second was to provide shelter hotels for DV victims. The third was to advertise the use of an emergency number through which a DV report is sent with a simple SMS [26]. The importance of preventing DV is a crucial aspect that was underestimated in the early stages of the COVID-19 pandemic. The weakest people, especially women, children, and the elderly, were the people most at risk and, in the initial phase of lockdown, they were not sufficiently protected by governments. In the future, it is hoped that the same mistakes will not be made and that these categories will be protected [27].

Several types of scams and fraud have been reported: cybercriminals are capitalizing on the anxieties and fears triggered by the pandemic, using malware, such as viruses, worms, trojan horses, ransomware, and spyware, to invade, damage, steal or delete personal data on personal computers. In addition to these types of online scams, there has been a surge in fake or inappropriate drugs and vaccines and medical equipment sold at a very high price [28,29]. Moreover, social isolation and decreased mobility combined with COVID-19 containment policies have inevitably increased stress levels, reducing access to social support services and increasing crime such as child abuse, domestic violence and substance abuse [30,31,32,33]. It was immediately thought that the COVID-19 pandemic, and especially lockdown, could negatively affect drug addiction, and in general the increase in the use/abuse of drugs [34]. In fact, drug addicts are among the populations at risk of contracting a severe SARS-CoV-2 infection as they are weak subjects. In fact, there was an increase in methadone overdoses at first, and pharmacies reported an increase in the production of fake medical prescriptions [35,36]. Furthermore, during lockdown, a vicious cycle was created characterized by the fact that substance abuse (such as opioids) increased the mortality of a COVID-19 related respiratory disease and, at the same time, preventive measures of COVID-19 (such as quarantine) increased the risk of drug abuse [37]. In fact, quarantine brought increased stress, irritability, boredom, sadness, and anger, and triggered the relapse of many ex-addicts. Indeed, drug addiction increased the likelihood of death, use of mechanical ventilation, and hospitalization. Even the symptoms of abstinence were overlooked due to the involvement of the entire health system in COVID-19, putting aside the rest of the diseases, especially those coming from substances of abuse [38]. The risk of substance abuse side effects during the pandemic was also linked to the fact that drug addicts were unable to reach treatment or implement appropriate detoxification treatments [39]. The ban on leaving the house, accessing outdoor spaces, led to a worsening of the drug addiction control of these patients [40]. Smart working also influenced the increase in alcohol consumption during the pandemic. Paradoxically, workers cannot have access to alcoholic beverages during working hours; however, working at home could run the risk of being drunk during smart working [41]. Several strategies were used to address the rise in drug abuse during the COVID-19 pandemic. One hypothesis was to improve telemedicine services, which were able to guarantee support to people through the prescription of drugs and/or laboratory tests. Another hypothesis was to implement “home hospitalization”, with the prescription of home care in order to reduce the number of hospital visits. An important aspect could also be to implement psychiatric counseling services, in order not to exile people with disorders from substance abuse and to offer a service at all times [42].

However, the drug trafficking trade underwent a drastic change during lockdown. Hence, the reduction in international travel led to a reduction in the trade in these drugs as well [43]. Finally, in this context, the work of the police services has also changed profoundly: priorities have become public order control activities, with a large part of the resources allocated to quarantine controls, imposing social distance, and implementing border controls [44]. Another important development was the redistribution of police personnel in cities to monitor the observance of the restrictive measures to contain the pandemic [45]. The policing of cities has left rural areas exposed to different types of crime [46]. In fact, this is another important change in crime management during the pandemic period [47].

### 2.2. Impact of Autopsy Practice on COVID-19 and Vice-Versa

Autopsy has always played an essential role not only in the forensic field, but also in clinical practice [48]. Different studies remark on the pivotal role of the forensic pathologist in gaining information about unknown diseases, and highlighting the importance of a multidisciplinary approach [49,50]. Nevertheless, the initial discouragement of autopsies in the early phase of the pandemic generated a real lockdown of science, not allowing researchers to find useful elements in the diagnosis and treatment of COVID-19 [51].

One of the most important problems related to the COVID-19 autopsy has been the recommendations of the scientific community to perform a safe autopsy [52]. An important issue concerning autopsy practice is related to the technical specifications recommended for post-mortem investigations, with particular attention to ventilation devices. The suggestion to have at least two zones—a “clean zone” and a “dirty zone”—to which an additional “intermediate zone” for the removal of protective clothing after the autopsy could be added, has posed serious difficulties related to space [53]. Furthermore, considering that SARS-CoV-2 has been classified as a Hazard Group 3 (HG3) pathogen, it has been recommended that autopsies should be performed in airborne infection isolation rooms (AIIR), providing negative pressure with respect to the surrounding areas [54]. If such autopsy rooms are not available, it has been recommended to carry out air exchange (a minimum number of 6 changes per hour); in addition, it has been suggested that air should be exhausted to the outside or through a high-efficiency particulate aerosol (HEPA) filter. Finally, there were further suggestions regarding the equipment dedicated to COVID-19 autopsies, suggesting the use of an oscillating or manual saw, antiviral disinfectants, specific devices for decontamination by fumigation (optimally), as well as the recommendation to minimize the number of people in the autopsy room [55]. Undoubtedly, adherence to these recommendations has contributed substantially to reducing the number of autopsies.

Despite these difficulties, the pivotal role of autopsy practice in the definition of SARS-CoV-2 physiopathology is now recognized. In fact, the first study published on COVID-19 autopsies showed deep vein thrombosis in 7 out of 12 patients and pulmonary embolism causing death in 4 of them. Histologically, the most common pulmonary findings were those of diffuse alveolar damage (DAD), hyaline membranes, activated pneumocytes, microvascular thromboembolism, and interstitial edema [56]. Another study by Wang et al. [57] on pulmonary alterations of people who died from COVID-19 showed a massive alteration of type I alveolar epithelial cells and atypical hyperplasia, confirming the presence of heterogeneous inflammatory tissue [58]. In another paper, platelet microthrombi were observed in 84% of subjects with immunohistochemical positivity to CD61 [59]. Similar results were also found by Fox et al. [60], and by Ackermann et al. [61]. Obviously, vascular endothelial inflammation was a common COVID-19 related finding [62,63,64].

Thanks to autopsy practice, it has been found that SARS-CoV-2 may be found in other organs. The brain represents a vulnerable organ to SARS-CoV-2 infection. The most common brain damage from COVID-19 infection was reported in a recent systematic review [65,66,67,68].

In the heart, SARS-CoV-2 infection also causes significant changes [69,70,71,72].

Concerning the liver, different hepatic histopathological changes related to COVID-19 were described. [73,74].

The kidney is also often affected by SARS-CoV-2 infection [75,76].

**Figure 1:**
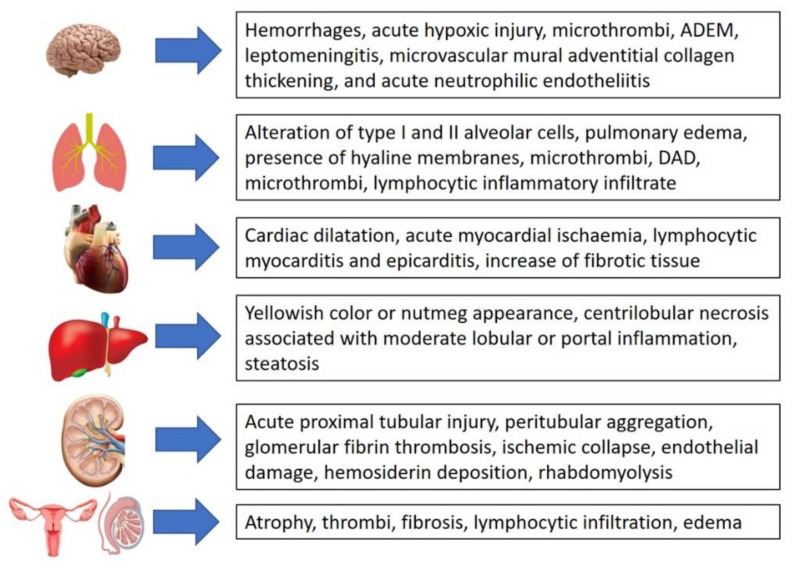
Summary of the main histopathological changes caused by COVID-19.

Figure 1 summarizes the major histopathological changes caused by COVID-19 in the different systems of the body.

Based on the discussed data, despite the fact that, at first, it was thought that the role of the forensic pathologist was marginal in the research and battle against COVID-19, it has been demonstrated that theirs is a crucial contribution in understanding not only the pathophysiology of the SARS-CoV-2 infection but also making an important contribution to therapy. The forensic world of the “dead” is complementary to that of clinical research on the “living”, traveling together for scientific research of any pathology [48,77,78].

### 2.3. Persistence of SARS-CoV-2 RNA in Post-Mortem Samples

Although different strategies have been developed to achieve the diagnosis of infection, to date, the gold standard method for diagnosing SARS-CoV-2 infection remains the detection of viral ribonucleic acid (RNA) in respiratory specimens, mainly obtained from nasal or oropharyngeal swabs [79].

Since several studies have shown the persistence of the virus on inanimate surfaces for up to 9 days [80,81], different guidelines were published worldwide for performing safety autopsies, suggesting several measures to limit the risk of infection for personnel involved in post-mortem investigations [82,83]. Typically, the guidelines suggest adherence to strict protocols, assuming that the virus could remain in deceased persons with replicative capacity, recommending the use of biosafety level (BSL) 3 protection standards in cases of suspected or confirmed COVID-19 cadaver examination.

The presence of SARS-CoV-2 RNA in post-mortem samples has been frequently investigated. It is possible to divide the studies into two groups, including in the first one all studies where the researchers evaluated the positivity of different samples to RT-PCR, and the second one, all studies where the authors evaluated the persistence of infectivity testing virus vitality.

In the first group, there are a great number of published studies: the positivity persistence to RT-PCR tests has been demonstrated in post-mortem samples ranging from a few hours after death [84] to 78 days after death [85]. Moreover, the positivity test has been demonstrated under different conditions: both in samples from corpses stored refrigerated (cold room) [86,87] and in exhumed corpses [88]. Nevertheless, it has been demonstrated too, that by applying specific procedures during the autopsy, infection risks may be minimized for the personnel involved in post-mortem investigations [89].

The second group includes all studies that analyzed the possibility that the virus preserved its possibility to infect a subject several days after death. Despite many articles demonstrating the persistence of SARS-CoV-2 RNA in post-mortem specimens, the possibility that the virus retains replicative capacity remains to be proven. To date, only a few studies have been conducted concerning the viability of the virus and the infectivity hazard to the personnel involved in COVID-19 corpse management [90,91]. Recent data demonstrated that a few hours (12 h) after death, the corpses of subjects who died with/from COVID-19 may be considered noninfectious.

Although these data should be confirmed with other studies, in support of the theory that corpses may be considered safe a few hours after death there are the general data about infection risks: despite the fact that the number of individuals who have died with or from COVID-19 is considerably high, there is no published report about infections carried by cadavers transmitted to forensic pathologists, technicians, or mortuary workers.

### 2.4. Forensic Personnel Activities during the SARS-CoV-2 Pandemic

In the pandemic period, the activities and planning of forensic laboratories underwent major changes, adapting them quickly to both workforce modification (for example to ensure social distancing) and the new demands related to crime modification. In the same manner, forensic personnel are called on to adapt their work habits to new work scenarios.

In this context, different forensic activities, such as Crime Scene Investigation (CSI) activities, forensic laboratory activities such as DNA testing, forensic examinations of abuse victims (in particular in cases involving minors), and autopsy methodologies have undergone inevitable changes. In the pandemic scenario, it became clear that all personnel involved in these activities were exposed to a high risk of infection [92].

In the first phase of infection, the Centers for Disease Control and Prevention (CDC) published guidelines to support the activities of law enforcement personnel. The last update of these guidelines is dated about one 1 year ago (6 November 2020) [93]. Subsequently, the guidelines were updated by the Occupational Safety and Health Administration (OSHA) with the aim of assisting employers and workers in forensic sciences to identify COVID-19 exposure risks, helping them to take appropriate steps to prevent exposure and infection [94]. In order to contain the risks of infection, it is important to consider a variety of factors such as the physical environment of the workplace (CSI operators must consider every crime scene as a “hot zone”), the type of work activity (for example CSI, post-mortem activities, laboratory tests), the health and/or vaccination status of workers (in this regard, forensic personnel should be fully vaccinated), the ability of workers to wear adequate personal protective equipment (PPE) and abide by current CDC guidelines. Moreover, it is important to limit close contact (within 6 feet for a total of 15 min or more over a 24-h period) with other people. Another important step is related to the treatment of non-disposable materials used during forensic activities: these materials should be sterilized at the end of each step, following the relative recommendations [95].

Finally, special considerations should be made for laboratory personnel as well as the required infrastructure for forensic laboratories. Considering that the guidelines recommended a BLS2 laboratory, this has increased the costs for law enforcement agencies and forensic institutions. In the same way, all personnel should be involved in specific training, both in biosafety practices and in the modification of well-defined protocols in order to prevent/contain hazardous risks of biological samples [96].

### 2.5. The Global Vaccination Program and Forensic Sciences

The COVID-19 pandemic caused a major global health, social, and economic crisis. At the end of December 2020, the approval of the first vaccine started the global vaccination program, which restored minimal confidence in the population [97]. The scientific community is convinced that, without a broad and comprehensive vaccination program, it will be impossible to overcome COVID-19. However, following some severe adverse events, a growing lack of confidence developed in many European countries, slowing down the worldwide vaccination program [98,99]. Similar to the case of the definition of the main pathophysiological aspects of the SARS-CoV-2 infection, post-mortem investigations have been instrumental in identifying the cause of death in fatal cases following vaccine administration [100,101,102,103]. Autopsy has also become crucial in establishing the causality relationship between vaccine administration and fatal adverse events [104]. Undoubtedly, the contribution of forensic science has been pivotal in restoring confidence in the vaccination program.

However, medico-legal aspects linked to the overall vaccination campaign in terms of medical professional liability, informed consent, and vaccination obligation are yet to be clarified [105]. Given that each country has adopted different measures to promote the vaccination campaign and different regulations, it is very hard to depict a complete overview. For example, these aspects are very different in relevance if vaccination is compulsory (for example, for healthcare professionals) or not. Complete and detailed information and reliance on scientific research are essential to understand the great importance of the vaccination campaign [106]. From a legal point of view, we must avoid blaming health professionals for side effects. At the same time, the scientific community is called on to protect the population by ensuring that indications, guidelines, and an appropriate method of administration are respected. On the other hand, from the point of view of civil law, it is correct to guarantee full protection in those rare cases where vaccine administration is linked to adverse events. All these aspects should be focused on in a specific literature review.

### 2.6. Forensic Undergraduate Education during and after the Imposed COVID-19 Lockdown

An important question strictly linked to the COVID-19 pandemic is related to forensic undergraduate education. All countermeasures to contain the risk of infection have limited university education/training. Education officials have been forced to cancel classes and close the doors to campuses across the world in response to the growing coronavirus outbreak. Social distancing and restrictive movement policies have significantly disturbed traditional educational practices [107]. To mitigate the adverse effects of restriction policies, several institutes have introduced modern technologies, developing virtual and/or remote laboratories, presenting an opening to explore creative ways of using technological solutions to form digitally generated forensic environments. Despite the fact that several universities have adopted alternative initiatives such as online sessions or using pre-recorded materials that could be considered valid for other degrees, practice activities are indispensable for forensic science. The CSI experience and autopsy practice cannot be substituted by videos, even if current technologies (i.e., 3D models produced by photogrammetry) may be useful as a support for autopsy and forensic pathology education [108]. In the same way, practical laboratory activities are necessary both for dexterity and to develop critical thinking [109]. Nevertheless, in a recent report based on student feedback, alternative lessons were evaluated positively, suggesting that in the near future they could be a valid support in forensic student education [110]. It is important to note that these initiatives have been very limited worldwide, considering that the use of virtual realities in forensic science is not diffused both in high and low-income countries.

In light of these considerations, it is important to preserve the classical practical lesson, using innovative instruments (such as video lessons) to support traditional practice.

### 2.7. The Medico-Legal Implications in Medical Malpractice Claims during the COVID-19 Pandemic

Medical negligence litigation has always been a controversial topic; however, the debate became even more heated during the COVID-19 pandemic [111]. Indeed, from the very beginning, both retired doctors and newly graduated doctors were called into hospitals [112]. The recruitment of non-specialist doctors and newly graduated medical students has further favored the frequency of errors in medical practice and the increase in cases of malpractice claims. This has further increased potential litigation within hospitals. It was originally thought that some sort of “immunity” for healthcare workers during the pandemic should be provided [113,114]. In the United States, the American Medical Association (AMA) proposed that healthcare professionals working against SARS-CoV-2 infection be protected from any liability. Other legal shields have also been proposed in the UK and Italy. The principle on which the immunity hypothesis is based is that healthcare professionals are unable to provide an adequate level of assistance in emergency conditions, excluding cases of gross negligence or willful misconduct [115]. However, this goal is unethical and unfair as it prevents people from receiving correct compensation for medical malpractice [111].

Despite the initial praise of the population towards health professionals in the fight against COVID-19, there were subsequently important medico-legal repercussions also due to diseases not related to the pandemic [116]. For example, there have been many litigations for diagnostic delays of carcinomas, or even delays in follow-up and in hospital admission for this disease. In fact, during the pandemic, many hospital departments of various kinds such as oncology, internal medicine, cardiology, and gynecology were transformed into COVID departments. Hence, hospital admission for other illnesses was drastically reduced, increasing subsequent medico-legal litigation related to malpractice claims [117]. For example, in an Italian hospital, there was an increase in medical-legal disputes due to delays in treatment, lack of hospitalization, and lack of health care for non-autonomous patients [118].

According to a recent systematic review, the hospital-acquired SARS-CoV-2 infection rate is 12–15% [119]. From a medico-legal point of view, this possibility of infection could be due, on the one hand, to an incorrect use of anti-COVID-19 measures such as social distancing, and PPE, and on the other hand to an inevitable risk due to the pandemic.

A good way to try to reduce malpractice litigation is to adopt a transparency policy within hospitals, and correct compilation of medical records. The medical record is of crucial importance for the prevention of hospital-acquired infections (HAI), regardless of COVID-19. Transcribing in the medical record the disinfection of the environments, the sterilization of surgical instruments, the sterilization of the operating field, and the appropriate use of PPE plays a crucial role in preventing malpractice litigation, also in the field of vaccinations. Transparency, communication with relatives and patients, trust between them and healthcare professionals play another fundamental role in preventing the risk of COVID-19 infection within hospitals [120]. Another interesting aspect is to encourage telemedicine in order to guarantee care for people who do not want to, or cannot, access hospitals, continue to perform follow-up, as well as avoiding diagnostic delays, and decreasing the correlated HAI due to COVID-19. However, telemedicine must always be used as a support for traditional medicine without ever replacing it [121].

## 3. Discussion

Responding to the COVID-19 pandemic, governments around the world adopted measures to contain the deadly spread of the virus, radically changing human habits [4,122]. Forensic sciences also had to adapt rapidly to the so-called COVID-19 era. In fact, forensic scientists and laboratory personnel had to rapidly modify their protocols and improve their training to quickly adapt to the operational changes imposed by the pandemic. Indeed, because of their profession, like other healthcare professionals, forensic laboratory staff were exposed to a high occupational risk for COVID-19 infection.

This narrative review aimed to provide an up-to-date overview on the different issues concerning COVID-19, analyzing the state of art about: the influence of external factors on forensic activities, the impact of autopsy practice on COVID-19 and vice-versa, the persistence of SARS-CoV-2 RNA in post-mortem samples, forensic personnel activities during the SARS-CoV-2 pandemic, the global vaccination program and forensic sciences, forensic undergraduate education during and after the COVID-19 imposed lockdown, and the medico-legal implications in medical malpractice claims during the COVID-19 pandemic.

As previously discussed, in some countries, the police have become a public health service, informing the public about isolation restrictions; in other cases, they have been used to implement coercive actions to disperse groups and manage risks. In other countries, the police have played an even larger role at times, including transporting and distributing essential supplies to remote communities to ensure that movement restrictions became feasible [44,45]. In this scenario, control in crime prevention has radically diminished. Similarly, the restrictive measures put in place to contain the spread of the COVID-19 infection have inevitably reduced some crimes (theft, robbery, street violence), although inevitably others (especially domestic violence and computer crime) have increased [10]. Nevertheless, it has not always been possible to respond adequately to the rapid change in the type of crimes committed: it is certainly desirable that in the future social support services to prevent the different types of domestic violence (on minors, women.) will be quickly reintroduced and that the monitoring and identification of cybercrimes will be improved. Social supports will be indispensable considering that the economic crisis related to the spread of the infection has increased unemployment.

Another aspect that has been strongly affected by the COVID-19 pandemic is related to the use of substances of abuse. In the last decade, globalization, the spread of online markets, and new technologies have led to the diffusion of new psychoactive substances, as well as the possibility of easily finding legal drugs used for hedonistic purposes. Obviously, the use/abuse of these substances has also increased in relation to the psycho-physical stress induced by the different measures taken by governments to reduce the spread of infection [43]. During the COVID-19 pandemic, mental health concerns assumed a clinical relevance, although it is not yet clear whether the etiology of the neurological and psychiatric symptoms observed in patients with COVID-19 were attributable to the virus itself, or alternatively, to the stress related to a pandemic or to pharmacological treatment [123]. At the same time, the search for effective drugs against COVID-19, as well as the difficulty for developing countries to implement effective vaccination campaigns, has meant that many people have resorted to the online drug market, buying dangerous substances and/or counterfeit drugs [124]. Based on the data discussed, it is worth highlighting the need to invest in developing and maintaining strong early warning and response systems for new psychoactive substances and licit and illicit drugs to protect public health.

Although it is well-known that autopsy represents the gold standard method for understanding the pathophysiological aspects of unknown diseases [50,125], during the COVID-19 pandemic there have been undoubted delays in performing autopsies on subjects who died with/from COVID-19. Taking a cautious approach to limit the risk of spreading the infection, only minimally invasive autopsies were initially performed [126]. Several weeks later, full autopsies were performed again, playing a vital role in understanding the pathophysiology of the SARS-CoV-2 infection [48,77,78]. Autopsy practice is closely linked to one of the major concerns of COVID-19 that remains not fully resolved: the safety of handling cadavers. The crucial aspect is the persistence of the virus in a deceased body. Assuming that the virus may be detected several days after death in the bodies of individuals who have died with/from COVID-19, different guidelines have been published in order to perform post-mortem investigations safely. However, although several studies have been published demonstrating the possibility to have a molecular positive swab test even in exhumed corpses, the viability of the virus, and thus the infectivity of a body, has been investigated only in few studies [85]. As previously discussed, based on a very recent study, the COVID-19 corpse may be considered safe a few hours after death. These data seem to be confirmed by the absence of evidence of infection from cadavers, also in consideration of the great number of COVID-19 deaths worldwide [90]. Another important aspect that supports this theory is related to corpse management of those who died from other causes (road accidents, suicides, deaths at work), because they may be asymptomatic individuals. This category includes individuals who are SARS-CoV-2 positive but have no clinical or radiological manifestations of COVID-19 [127]. Personnel involved in corpse management (mortuary staff, first aid personnel, pathologists) did not always apply the measures prescribed for handling COVID-19 corpses; despite this, no cases of infection related to this type of practice have been reported [90]. This aspect is strictly related to forensic personnel activities: all investigations should be considered at high risk of infection, taking the right countermeasures [89]. Undoubtfully, these aspects, on the one hand, have slowed down forensic operations, and on the other hand, have raised costs.

In light of these considerations, as remarked in this review, the definition of these aspects remains crucial in order to ensure a possible update of the guidelines in the handling of COVID-19 corpses. Clarifying these important aspects could be beneficial not only for professional operators but for forensic undergraduate education too [110]. Despite the fact that during the pandemic period, the use of modern technologies has been adopted with positive results, several practical activities, such as autopsy, should be considered mandatory in forensic training [109].

Finally, another crucial aspect during the COVID-19 pandemic is related to the health care system. Many healthcare workers have worked in unfamiliar environments, in several cases, carrying out new tasks, trying to cope with an unprecedented workload in a general context with a lack of knowledge about the virus. This scenario exposed healthcare workers to an increase in complaints about the treatment provided in these circumstances, and conflicting arguments about how these should be handled within the criminal, civil and regulatory systems [113]. In addition, with the start of the vaccination campaign, new responsibilities arose for health workers employed in these activities, with new risks of liability [128]. At the same time, the COVID-19 emergency has enabled the implementation of extraordinary and previously little-known measures such as telemedicine, which in the near future could be useful, especially to ensure public health cost containment and timely support for patients.

## 4. Conclusions

The COVID-19 pandemic has greatly influenced different aspects of human life, and accordingly the practical activities of forensic sciences that are defined as multidisciplinary, involving different expertise. Indeed, the activities are very different, including CSI, external examination, autopsy, genetic, and toxicological examinations of tissues and/or biological fluids. At the same time, forensic professionals may have direct contact with live subjects, such as in the case of abuse victims (involving in some cases children), collecting biological samples from suspects, or visiting subjects in the case of physical examinations. Moreover, different external factors, such as the modifications of criminal activities, have greatly influenced forensic practice. On the one hand, during the pandemic period, the number of crimes has inevitably decreased (particularly for homicides and theft). On the other hand, it should be noted that medico-legal disputes have largely increased due to complaints about deaths that occurred in hospitals and in nursing homes related to the COVID-19 disease. Furthermore, the increased psychological stress negatively influenced domestic violence crimes, and accordingly the medico-legal activities. In this way psychological support should be supplied for all subjects, particularly in cases of adoption of severe social restrictions. This may mitigate the adverse effects on mental health. In the same way, this support should be guaranteed both to enforce polices and for health care: these categories have particularly suffered the great impact of COVID-19. It is also necessary that governments adopt financial support for these categories, providing the essential tools such as PPE, to guarantee a safe working environment, and sustaining research activities in these fields. Particularly, special support should be given to contain medico-legal implications in medical malpractice claims during the COVID-19 pandemic. The last message is reserved for forensic education: the COVID-19 crisis has highlighted the use of alternative activities to train the professionals of tomorrow, demonstrating the importance of practical activities that cannot be not substituted with modern technologies.

## Supplementary Materials

The following are available online at https://www.mdpi.com/article/10.3390/healthcare10020319/s1, Figure S1: Flow diagram of the literature search and study selection for this review (PRISMA flow chart).
