# Supplementary material for: The Impact of the COVID-19 Pandemic on the Practice of Forensic Medicine: An Overview
Source: Healthcare (Basel). 2022 Feb 8;10(2):319. doi: 10.3390/healthcare10020319 (PMC8871677; doi:10.3390/healthcare10020319)
Supplement: Supplementary file 1 [file healthcare-10-00319-s001.zip › healthcare-1540540-supplementary.pdf]

**Supplementary material, manuscript title:" The impact of the COVID-19 pandemic on the practice of forensic medicine: an overview"**

## S1 Methods

### S1.1 Search Strategy

The Preferred Reporting Items for Systematic Reviews and Meta-Analyses Statement (PRISMA) recommendations were applied to perform this systematic review [1]. We searched all publications related to SARS-CoV-2/COVID-19 and the following thematic: - the impact of COVID-19 on criminal investigations, with a focus on domestic violence; - the use of new psychoactive substances during the COVID-19 pandemic; - the impact of COVID -19 on autopsy practice; - the persistence of SARS-CoV-2 RNA in post-mortem samples; - forensic personnel activities during the SARS-CoV-2 pandemic; - forensic undergraduate education during and after the imposed COVID-19 lockdown; and the medico-legal implications in medical malpractice claims during the COVID-19 pandemic. To perform this review, the following databases were searched: Scopus, Medline (via PubMed), and Web of Science. All studies published up to 20 January 2022 were searched without language restrictions by three independent reviewers. Searched medical subject headings (MeSH) were: "COVID-19", "SARS-CoV-2", and "criminal investigations", "domestic violence", "new psychoactive substances", "autopsy practice", "vaccine adverse effects", "RNA persistence", "forensic activities", "forensic undergraduate education", "medical malpractice claims".

### S1.2 Study Selection

The retrieved studies were first reviewed by three independent authors (FS, MS, and ME) based on the title and abstract, all unrelated publications were removed and the full texts of the remaining articles were fully reviewed. Then, two independent reviewers (ES and NDN) judged potentially eligible articles, and disagreements were resolved by discussion and for each article a consensus was reached.

### S1.3. Eligibility, Inclusion, and Exclusion Criteria

The following predetermined conditions had to be met for studies to be considered for inclusion in this review. For initial screening, all studies focusing on the review's thematic were included in the review; English language was an inclusion criterion. Any papers that focused on a different theme with minor considerations about the thematic, review, letters, note, editorial, conference paper, short survey, book chapter, conference review, erratum, book, and retracted were excluded from this review.

121 papers met the narrative review inclusion criteria (Figure 1).

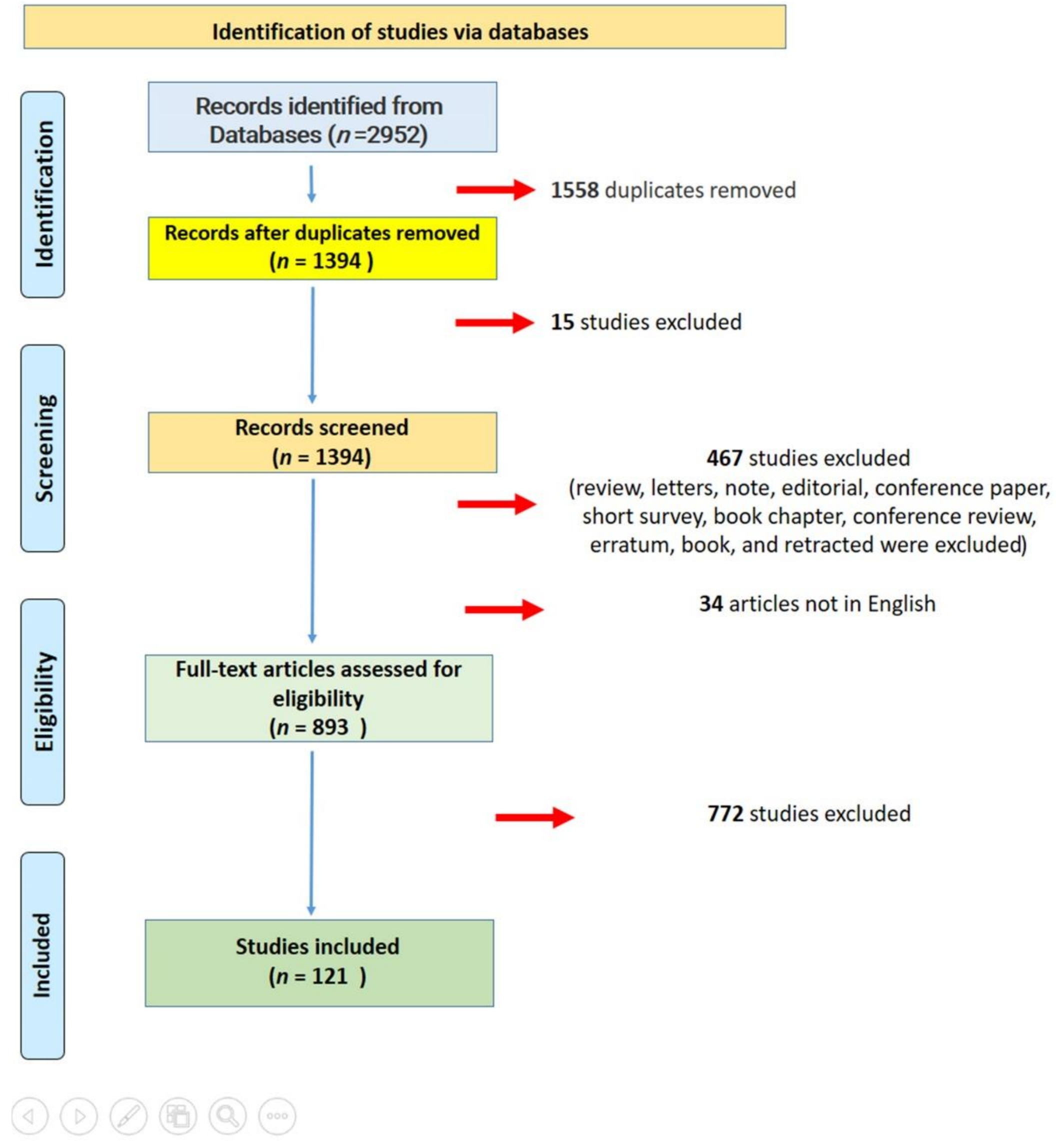


**Figure S1.** Flow diagram of the literature search and study selection for this review (PRISMA flow chart).

## References

1. Moher, D.; Shamseer, L.; Clarke, M.; Ghersi, D.; Liberati, A.; Petticrew, M.; Shekelle, P.; Stewart, L.A. Preferred reporting items for systematic review and meta-analysis protocols (PRISMA-P) 2015 statement. *Syst. Rev.* **2015**, *4*, 1.
